# Impact of SARS-CoV-2 Omicron infection on late pregnancy: an analysis of clinical characteristics, pathology of placenta and outcomes in a case-control study

**DOI:** 10.1515/med-2026-1444

**Published:** 2026-08-03

**Authors:** Heping Zhang, Hongliang Xu, Shuguang Zhou, Yong Chen, Qin Wang, Caixia Zhao, Man Tang, Weiqin Zhang

**Affiliations:** Department of Pathology, Anhui Women and Children’s Medical Center, Hefei Maternity and Child Health Hospital, Anhui Medical University, Hefei, People’s Republic of China; Department of Gynecology and Obstetrics, Anhui Maternity and Child Health Hospital, Hefei, People’s Republic of China

**Keywords:** Omicron, placenta, fetal distress, SARS-CoV-2, outcome

## Abstract

**Objectives:**

This study analyzes clinical features and placental pathology of pregnant women infected with SARS-CoV-2 Omicron variants and conducts a two-year follow-up to assess childbirth risks and newborn health outcomes.

**Methods:**

Retrospective analysis of 46 Omicron-infected pregnant women (Omicron group) and 96 uninfected pregnant women (Control group).

**Results:**

Omicron group exhibited higher risk of fetal distress rate, lower leukocyte and lymphocyte counts, and higher C-reactive protein levels compared to the control group. Most infected women (93.48 %) were symptomatic, with fever being the most common symptom. Histologically, acute maternal and fetal inflammatory response were observed in 28.26 and 8.70 % of the Omicron group, but no significant difference from the control group. Maternal vascular malperfusion (50.00 %) and fetal vascular malperfusion (4.34 %) were present in the Omicron group at rates comparable to the controls. Among the 39.13 % of newborns tested for SARS-CoV-2 within three days of birth, 33.33 % tseted positive. Of these positive neonates, 83.33 % were symptomatic, universally presenting with fever, and one developed pneumonia.

**Conclusions:**

Placental pathological findings were non-specific to the Omicron variant. Notably, the two-year follow-up indicated that neither the Omicron-infected mothers nor their newborns experienced long-term adverse health outcomes.

## Strengths and limitations of this study

### Strengths

This study provides valuable insights into the effects of SARS-CoV-2 Omicron infection during late pregnancy. The primary strengths of this research include:

1. **Comprehensive follow-up:** A significant strength is the two-year follow-up of both Omicron-infected mothers and their newborns, providing robust evidence regarding the absence of long-term adverse outcomes. This extended follow-up period is critical for assessing the lasting impact of perinatal infections.
2. **Detailed clinical and laboratory data:** The study meticulously collected and analyzed clinical data, including leukocyte, lymphocyte count and C-reactive protein (CRP) concentrations, providing objective measures of the maternal immune response differences between the Omicron and control groups.
3. **In-depth placental pathological analysis:** The inclusion of detailed placental pathology allowed for the identification of specific changes like Maternal Vascular Malperfusion (MVM) facilitating the hypothesis regarding the potential lack of placental invasiveness by the Omicron variant.

### Limitations

Despite its strengths, this study has some limitations that should be explicitly acknowledged:

1. **Lack of specific variant lineage information:** The absence of genomic sequencing prevents the precise identification of the infecting sub-strains.
2. **Sample size and model instability:** The retrospective design and small sample size result in extremely wide confidence intervals in our multivariable logistic regression, reflecting potential model instability. The non-universal, symptom-based screening strategy for newborns introduces a significant selection bias, limiting the precise assessment of vertical transmission.
3. **Confounding by maternal vaccination:** All participants received three doses of the COVID-19 vaccine. While this controls for vaccination status, it limits generalizability, as the mild symptoms and favorable outcomes are likely mediated by vaccine-induced immunity and cannot be extrapolated to unvaccinated populations.

## Introduction

Globally, the Coronavirus disease 2019 (COVID-19), caused by Severe Acute Respiratory Syndrome Coronavirus (SARS-CoV-2), has infected more than 400 million individuals and resulted in over 4 million fatalities [1]. In China, the infection rates of SARS-CoV-2 were kept to a minimum due to the implementation of a stringent “Zero COVID-19” policy until December 2022. Following the relaxation of this policy, there has been a sharp increase in COVID-19 cases. The Technical Advisory Group on Virus Evolution (TAG-VE) identified Omicron variants BA.5.2 and BF.7 as the predominant strains circulating in China [2].

Pregnant women represent a demographic particularly susceptible to SARS-CoV-2 infection, with outcomes ranging from asymptomatic cases to upper respiratory tract infections and more severe manifestations [3]. While the majority of pregnant women with SARS-CoV-2 exhibit mild symptoms [3], [4], [5], there is evidence suggesting an elevated risk of progression to severe disease in this population [6], [7], [8]. Beyond clinical symptoms, infection during pregnancy is known to trigger significant pathophysiological changes, most notably a disturbance in the body’s redox balance. Studies confirm that infected pregnant women and their newborns exhibit significantly elevated levels of systemic oxidative stress, as evidenced by increased levels of biomarkers like thiobarbituric acid-reactive substances (TBARS), an index of lipid peroxidation [9].

The placenta, a transient yet critical organ for fetal development, is essential for nutrient transfer from mother to child but becomes redundant post-delivery. This organ represents a potential route for SARS-CoV-2 transmission to the newborn. Although previous studies have not detected SARS-CoV-2 variants within placental tissues [10], recent immunohistochemical analyses have successfully identified the presence of the SARS-CoV-2 spike protein within placental tissues, which is associated with a pronounced inflammatory response characterized by an increased infiltration of CD68+ macrophages. This localized inflammation is strongly correlated with systemic maternal and fetal oxidative stress levels and is linked to a significantly higher incidence of placental lesions, particularly Fetal Vascular Malperfusion (FVM). These pathological changes are not merely histological findings; they have direct clinical relevance. The level of spike protein expression in the placenta has been shown to correlate directly with adverse neonatal outcomes, such as a longer and more frequent need for care in the neonatal intensive care unit (NICU) [11].

Amidst the recent surge, our hospital, recognized as the premier obstetrics and gynecology facility in Anhui Province, observed a marked rise in COVID-19 infections among pregnant women. However, despite the prevalence of the Omicron variant, data specifically elucidating its impact on placental histopathology and long-term neonatal outcomes remain limited. We hypothesized that while Omicron infection in the third trimester would induce a maternal systemic inflammatory response, it would not result in severe specific placental malperfusion or long-term adverse health outcomes for the offspring. Consequently, our primary research question was whether SARS-CoV-2 Omicron infection independently increase the risk of specific placental histopathological abnormalities and adverse neonatal outcomes compared to uninfected controls. We conducted a study involving 142 pregnant women, including 46 Omicron-infected pregnant women and 96 non-infected controls. Our analysis encompassed clinical characteristics, laboratory findings, placental pathology, neonatal outcomes, and a two-year follow-up, providing valuable insights into the management and prognosis of COVID-19 in pregnant women.

## Methods

### Study population

**Figure 1:**
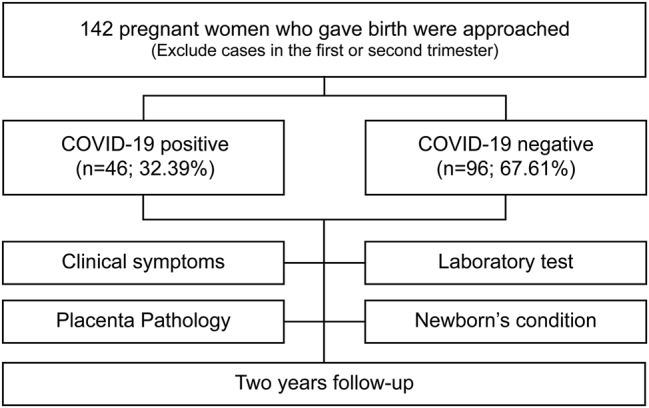
Women enrolled in the cohort at our hospital.

All participants are Asian residents of Anhui Province, China, who delivered their babies and submitted the placentas for pathological examination at our hospital. All included patients had received three dose of a COVID-19 vaccine (Beijing Institute of Biological Product Co., LTD, 0.5 mL/dose; or Sinovac Biotech Ltd., 0.5 mL/dose) and were in their third trimester of pregnancy; those in their first or second trimester were excluded. To ensure the integrity of the control group, all patients received SARS-CoV-2 PCR testing upon admission. The strict “Zero COVID-19” policy mandated continued serial testing, which effectively allowed us to eliminate asymptomatic cases from the Control group. A total of 142 women were enrolled: 46 in the Omicron group (aged 22–41 years) and 96 in the Control group (aged 21–39 years) (Figure 1). Clinical data collected included maternal age, gestational age, clinical symptoms of Omicron infection, and laboratory test results. Clinical characteristics were extracted directly from the patients’ medical records and the accompanying pathology application forms provided by the attending obstetrician or surgical team. Maternal recovery and neonatal conditions were monitored for two years via standardized telephone interviews and patient’s records during their regular physical examinations at our hospital.

### Laboratory tests

All blood samples were collected within 24 h after admission. Maternal peripheral blood samples were collected in ethylenediaminetetraacetic acid (EDTA) tubes. Laboratory tests included leukocyte count (reference range: 6–18 × 10^9^/L), lymphocyte count (reference range: 1.5–4.0 × 10^9^/L), and C-reactive protein (CRP) levels (reference range: 0–10 mg/L).

### Pathology

All placentas underwent routine pathological examination at our hospital, irrespective of the patient’s COVID-19 status. The examination was conducted by obstetrics and gynecology pathologists with subspecialty training, and placental lesions were classified according to the criteria established by the Amsterdam Placental Workshop Group Consensus Statement [12], specifically assessing for features of acute maternal/fetal inflammatory response, maternal vascular malperfusion (MVM), and fetal vascular malperfusion (FVM). Each placenta was sampled systematically, including two cross sections of the umbilical cord, a rolled section of the extraplacental membranes, one section from the border, and two full-thickness sections from the central region, along with any additional random sections taken as necessary. All the samples were fixed and processed using Leica HistoCore PELORIS 3 Premium Tissue Processing System (Model: 45.0001). The resulting FFPE blocks were sectioned at a thickness of 3 µm and subsequently stained with hematoxylin and eosin (H&E) using Leica HistoCore SPECTRA Workstation (Workstation Kit: 14051254355).

### SARS-CoV-2 Omicron variants detection

All pregnant women underwent SARS-CoV-2 PCR testing within 24 h after admission, and SARS-CoV-2 PCR testing was conducted for eighteen newborns born to positive pregnancy women after birth within 24 h. Following the end of the strict ‘Zero-COVID-19’ policy, PCR testing for newborns became reserved primarily for those exhibiting clinical symptoms of infection or those born to mothers with severe disease rather than a universal screening protocol. Detection of SARS-CoV-2 Omicron variants was carried out with the 2019-nCoV nucleic acid detection kits supplied by Wuhan EasyDiagnosis Biomedicine Co., Ltd and DAAN GENE Co., Ltd, China. Each specimen underwent simultaneous testing with kits from both providers. The kits target the nucleocapsid (N) gene and open reading frame (ORF) regions of SARS-CoV-2 Omicron variants. The cycle threshold (Ct) values were obtained from a Real-time fluorescent PCR instrument (Gentier 96R, Tianlong Technology, China), where a Ct value below 35 was considered indicative of SARS-CoV-2 Omicron variants infection.

### Statistical analysis

Data analysis was conducted using SPSS software, version 26.0 (IBM, Armonk, NY, USA). Comparative analyses of baseline demographic and clinical characteristics – including maternal age, gestational age, parity, and relevant maternal comorbidities – between COVID-19-positive maternities and control group were presented as counts and percentages. Comparisons were made using Student’s T-test for parametric data, and Fisher’s exact test for categorical variables. To control for potential confounding factors, a multivariable binary logistic regression analysis was performed to identify independent risk factors for fetal distress. The model included the study group (Omicron vs. Control) and baseline characteristics that showed statistical differences in the univariate analysis (specifically, scarred uterus and epilepsy). Results are presented as adjusted odds ratios (aOR) with 95 % confidence intervals (CI). A two-sided p-value <0.05 was considered statistically significant.

### Patient and public involvement

It was not appropriate or possible to involve patients or the public in the design, or conduct, or reporting, or dissemination plans of our research.

### Ethical approval

The study was conducted with approval from the institutional review board at Anhui Medical University (No. 83240052). This study was approved by the Institutional Review Board (IRB) at the Anhui Medical University.

### Informed consent

Patients consented to an informed consent process that was reviewed by the IRB.

All specimens were coded and handled according to the ethical guidelines described by Anhui Medical University and were reviewed and approved by the Anhui Medical University Ethics Committee.

## Results

### Clinical characteristics of Omicron-infected pregnant women

A total of 37 women (80.43 %) in the Omicron group and 79 women (82.29 %) in the control group were nulloparous. There was no statistically significant difference in the proportion of nulliparous women between the groups (p=0.79). The average and median gestational age at delivery was 38.7/39.4 weeks in Omicron group (ranges from 31.14 to 41 weeks) and 39.3/40 weeks (ranges from 32.29 to 41.29 weeks) in control group. (Table 1). Omicron-infected women were more likely to experience fetal distress compared to the control group (45.65 % vs. 3.13 %) (Table 2). After adjusting for the confounding effects of a scarred uterus and epilepsy using multivariable logistic regression, Omicron infection remained an independent risk factor for fetal distress (aOR=30.898; 95 % CI: 8.342–114.448; p<0.001). In the Omicron group, 43 (93.48 %) presented with clinical symptoms. Fever was the most common clinical symptom (42, 91.30 %), followed by sore throat, myalgia, or limb weakness (13, 28.26 %), diarrhea, chest pain, or chills (4, 8.70 %), chest tightness or dyspnea (2, 4.35 %), bloating, dizziness and fatigue (4, 8.70 %).

**Table 1:** Clinical characteristics of Omicron maternities and control group.

	Control (n=96)	Omicron group (n=46)	p-Value
Maternal age (years; mean and median)	29.6; 29	29.4; 29	0.97
Primiparity (n, %)	79 (82.29)	37 (80.43)	0.79
Gestational age at delivery (weeks; mean and median)	39.3; 40	38.7; 39.4	0.02
Birthweight (grams; mean and median)	3,351.77; 3,370	3,429.02; 3,435	0.33
Preterm delivery (<37 weeks; n, %)	6 (6.25)	6 (13.04)	0.008
Term delivery (37–41 weeks; n, %)	90 (93.75)	40 (86.96)	
Apgar score (1 min, 5 min; n, %)			0.30
4, 8	1 (1.04)	0	
5, 10	1 (1.04)	0	
6, 9	1 (1.04)	0	
8, 9	0	2 (4.35)	
8, 10	1 (1.04)	0	
9, 10	92 (95.83)	44 (95.65)	

**Table 2:** Details of pregnant women with pregnancy complications (n, %)^a^.

	Control (n=96)	Omicron group (n=46)	p-Value
Fetal distress	3 (3.13)	21 (45.65)	<0.001
Epilepsy	0	3 (6.52)	0.03
Hypothyroidism	9 (9.38)	1 (2.17)	0.11
Diabetes	11 (11.46)	1 (2.17)	0.054
Uterine fibroids	0	1 (2.17)	0.32
Uterine scar	2 (2.08)	5 (10.87)	0.04
Central placenta previa	1 (1.04)	0	0.68
Syphilis	1 (1.04)	0	0.68
Proteinuria	1 (1.04)	0	0.68
Intrahepatic cholestasis	4 (4.17)	0	0.21
Liver damage	1 (1.04)	0	0.68
High blood pressure	10 (10.42)	1 (2.17)	0.08
Preeclampsia	1 (1.04)	0	0.68
Intrauterine growth restriction	1 (1.04)	0	0.68
Hypoproteinemia	1 (1.04)	0	0.68
Giant baby	4 (4.17)	1 (2.17)	0.48
Anemia	2 (2.08)	0	0.46
Complete placenta previa	1 (1.04)	1 (2.17)	0.55
Placenta accreta	1 (1.04)	0	0.68
Oligohydramnios	1 (1.04)	1 (2.17)	0.55
Hepatitis B	1 (1.04)	0	0.68
Fever	0	2 (4.35)	0.10
Acute chorioamnionitis	0	3 (6.52)	0.03
Antepartum hemorrhage	0	1 (2.17)	0.32
Cephalopelvic disproportion	0	2 (4.35)	0.10

^a^Some patients have multiple complications.

### Laboratory test of control and Omicron-infected pregnant women

The mean leukocyte count in the Omicron group was significantly lower than in the control group (8.71 vs. 10.50 × 10^9^/L, p=0.009). Furthermore, 37 women (80.43 %) in the Omicron group exhibited a lower lymphocyte count compared to the control group (48, 50.00 %) (0.96 vs. 1.55 × 10^9^/L, p<0.001). In addition, elevated CRP levels were observed more frequently in the Omicron group (40, 86.96 %) than in the control group (36, 37.50 %, p<0.001). The average and median Ct values for SARS-CoV-2 were 26.71, 26.24 (N gene, Quartiles: 23.29, 26.24, 30.87) and 26.13, 25.89 (ORF, Quartiles: 22.82, 25.89, 29.93), suggesting that delivery did not significantly impact viral replication rates (Table 3).

**Table 3:** Laboratory and placental pathology of Omicron maternities and control group.

	Control (n=96)	Omicron group (n=46)	p-Value
**Laboratory** ^**a**^			
**Leukocyte ( × 10** ^**9**^ **/L)**	10.50	8.71	0.009
Normal	93 (96.87)	39 (84.78)	
Elevated (>18 × 10^9^/L)	1 (1.04)	0	
Decreased (<6 × 10^9^/L)	2 (2.08)	7 (15.22)	
**Lymphocytes ( × 10** ^**9**^ **/L)**	1.55	0.96	<0.001
Normal	47 (48.96)	9 (19.57)	
Elevated (>4 × 10^9^/L)	1 (1.04)	0	
Decreased (<1.5 × 10^9^/L)	48 (50.00)	37 (80.43)	
**CRP, mg/L**			<0.001
Normal	60 (62.50)	6 (13.04)	
Elevated (>10 mg/L)	36 (37.50)	40 (86.96)	
**Ct value of SARS-CoV-2**			
N	N/A	26.71	
ORF	N/A	26.13	
**Placenta**			
Weight (grams, mean&median)	505.85 (500)	488.48 (500)	0.480
Fetoplacental weight ratio (n, %)			0.528
<25th percentile	22 (22.92)	12 (26.09)	
25th –50th percentile	36 (37.50)	14 (30.43)	
51st –75th percentile	19 (19.79)	12 (26.09)	
>75th percentile	19 (19.79)	8 (17.39)	
Acute maternal inflammatory response^b^ (n, %)	30 (31.25)	13 (28.26)	0.256
Stage 1	21 (70.00)	10 (76.92)	
Stage 2	9 (30.00)	3 (23.08)	
Acute fetal inflammatory response^c^ (n, %)	3 (3.13)	4 (8.70)	0.061
MVM	53 (55.21)	23 (50.00)	0.227
FVM	2 (2.08)	2 (4.34)	0.751

^a^Reference value range of laboratory test. Leukocyte: 6–18 × 10^9^/L, Lymphocytes; 1.5–4 × 10^9^/L; CRP: 0–10 mg/L. ^b^Acute maternal inflammatory response includes Stage 1 (acute subchorionitis or chorionitis), Stage 2 (acute chorioamnionitis; polymorphonuclear leukocytes extend into fibrous chorion and/or amnion). ^c^Acute fetal inflammatory response includes Stage 1 (chorionic vasculitis or umbilical phlebitis), Stage 2 (involvement of the umbilical vein and one or more umbilical arteries). COVID-19, coronavirus disease 2019; N/A, not applicable. CRP, C-reactive protein; MVM, maternal vascular malperfusion; FVM, fetal vascular malperfusion; N, nucleocapsid gene; ORF, open reading frame.

### Placenta pathology of control and Omicron infected pregnant women

**Figure 2:**
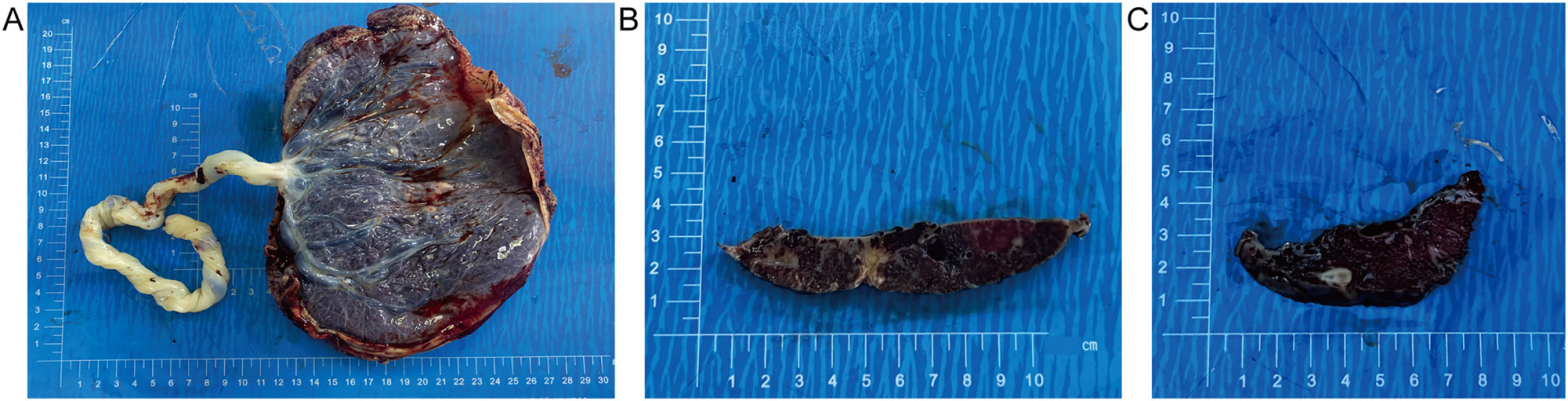
Gross features of the COVID-19 placenta. A) Marginal umbilical cord insertion, B) a gray-yellow infarct zone exists in the center of the placenta and C) a gray-white infarct zone exists in the paracenter of the placenta.

**Figure 3:**
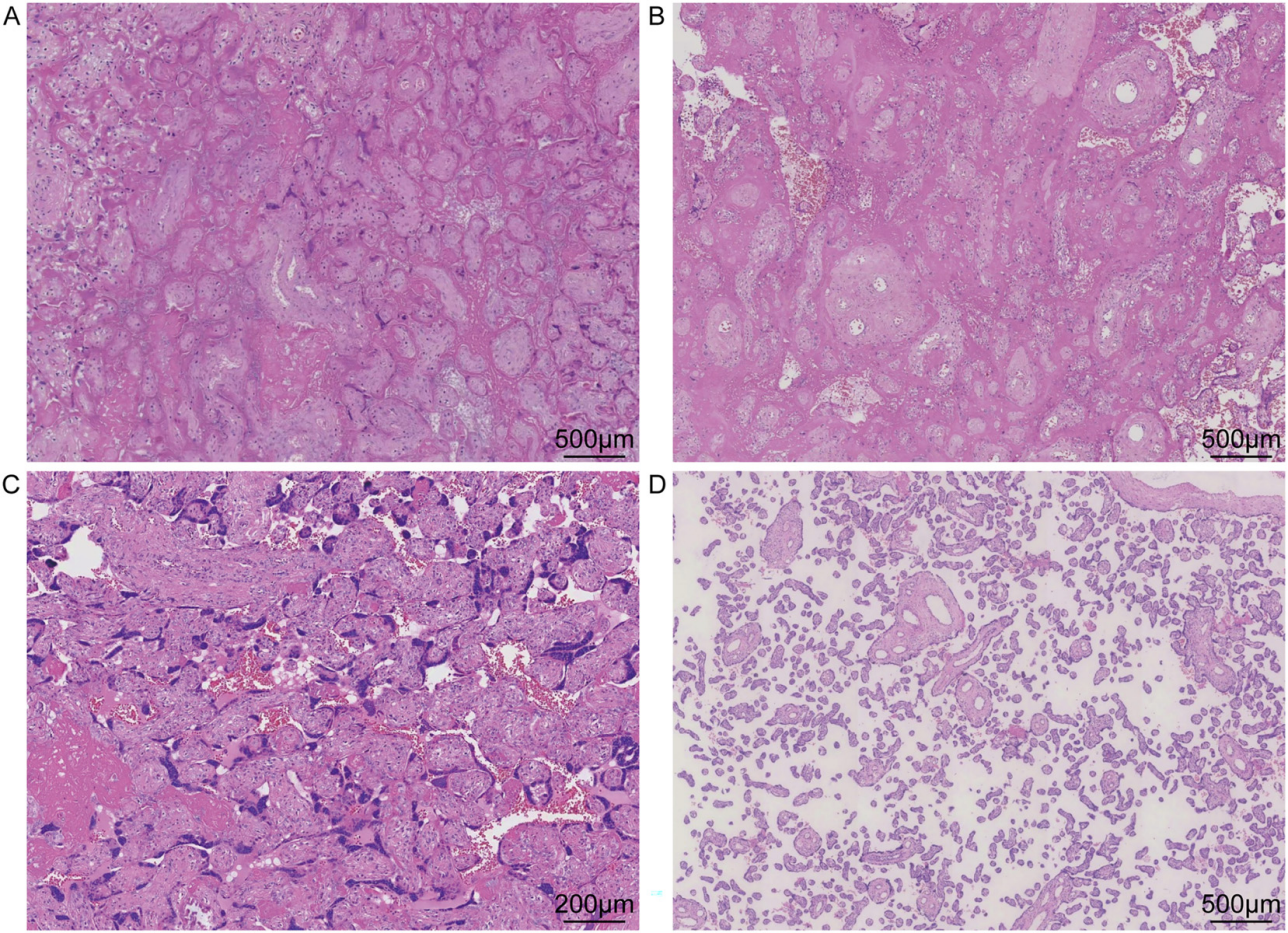
A–D) Maternal vascular malperfusion of the COVID-19 placenta. A) Infarcted. The villous tissue in the infarcted area loses its normal structure, showing the characteristics of coagulative necrosis. The tissue becomes solidified, cell structure is lost, cell details are obliterated, and cell nuclei disappear (magnification: × 40), B) fibrin deposition. Fibrin deposition could be observed between the villous (magnification: × 40), C) syncytial knots are increased. The number of syncytial knots were increased, which typically have hyperchromatic nuclei and condensed cytoplasm (magnification: × 100) and D). Distal villous hypoplasia. The villi are thin and relatively elongated appearing (magnification: × 40).

**Figure 4:**
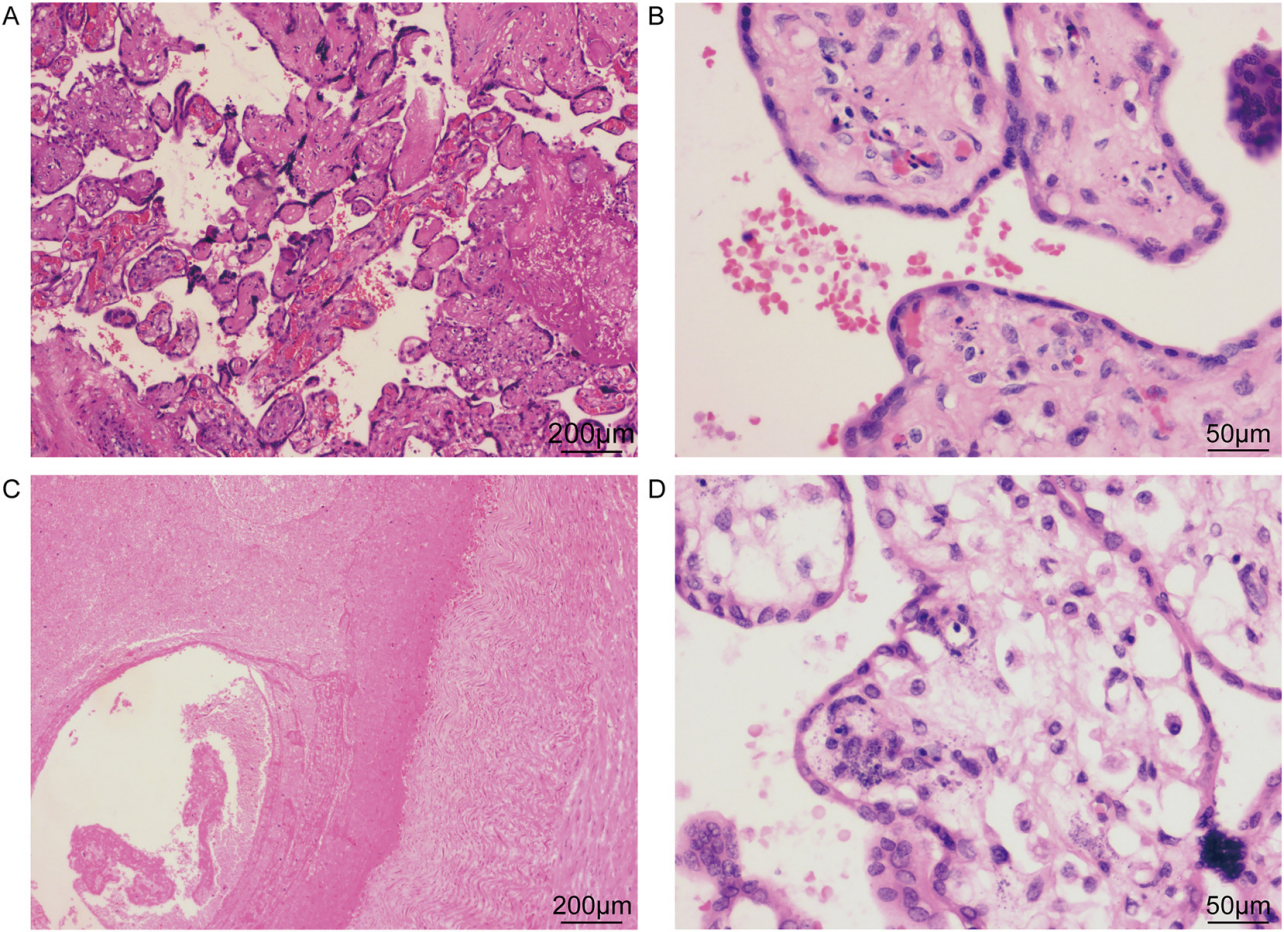
A–D) Fetal vascular malperfusion of the COVID-19 placenta. A) Avascular villi. The villi showing total loss of villous capillaries and bland hyaline fibrosis of the villous stroma (magnification: × 400), B) villous stromal karyorrhexis. Terminal villi showing karyorrhexis of fetal cells with preservation of surrounding trophoblast (magnification: × 100), C) thrombosis. Thrombosis could be observed in the umbilical vein (magnification: × 100) and D) villous edema. The villi are significantly enlarged, with uneven sizes and distorted shapes (magnification: × 400).

**Figure 5:**
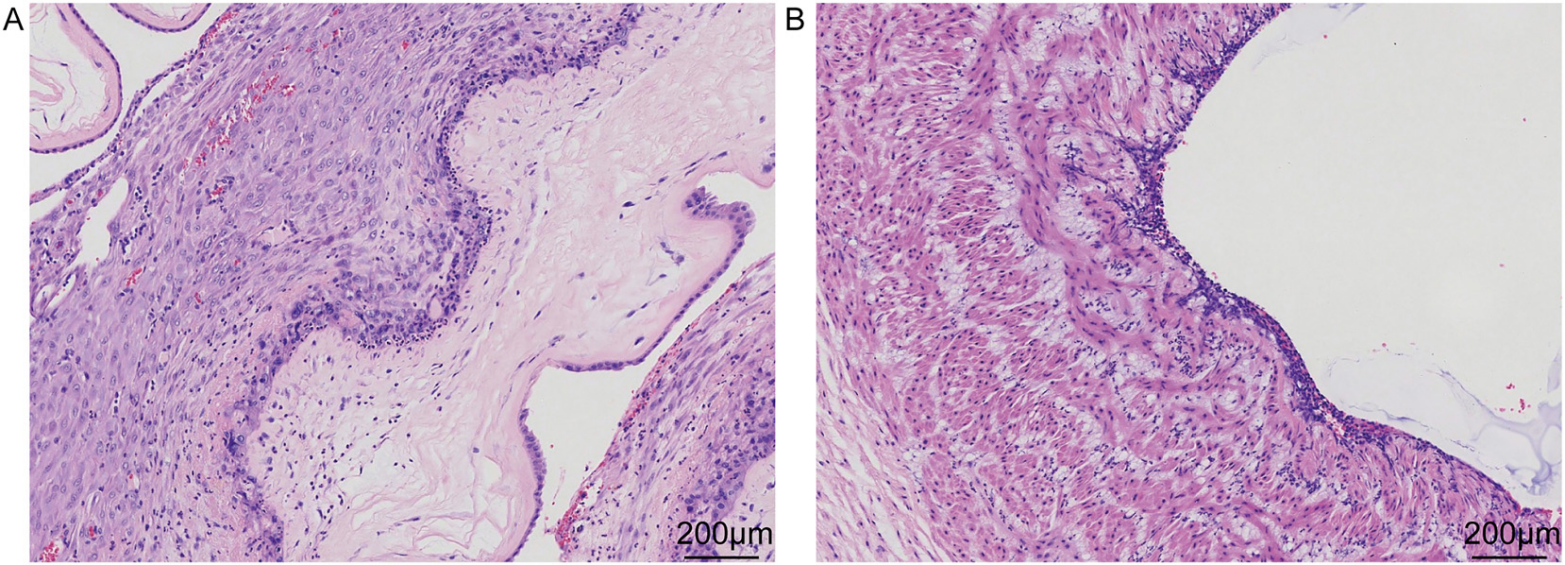
Maternal and fetal inflammatory responses of the COVID-19 placenta. A) Acute chorioamnionitis. Polymorphonuclear leukocytes extend into fibrous chorion and amnion (magnification: × 100) and B) acute umbilical phlebitis. Infiltration of neutrophils within the vessel wall (magnification: × 100).

The average and median placental weight were 488.48/500 g in the Omicron group (ranges from 200 to 760 g) and 505.85/500 g in the control group (ranges from 200 to 620 g). The fetoplacental weight ratio for both groups were concentrated within the 25 to 75th intervals. Gross examination revealed macroscopic abnormalities in 9 (19.57 %) placentas from the Omicron group, including marginal umbilical cord insertion and infarction (Figure 2A–C). Histologically, maternal vascular malperfusion (MVM), including infarcts, distal villous hypoplasia, accelerated villous maturation, was identified in 23 (50.00 %) in the Omicron group (Figure 3A–D). Fetal vascular malperfusion (FVM), including thrombosis, avascular villi, and villous stromal-vascular karyorrhexis, was found in 2 (4.34 %) cases (Figure 4A–D). These rates were similar to those observed in the control group (MVM, 53, 55.21 %; FVM, 2, 2.08 %). An acute maternal inflammatory response was present in 13 (28.26 %) cases in the Omicron group (Figure 5A and B), comprising 10 (76.92 %) stage 1 cases and 3 (23.08 %) stage 2 cases, with no significant differences compared to the control (30, 31.25 %). An acute fetal inflammatory response occurred in 4 (8.70 %) cases in the Omicron group, which was slightly higher but not significantly different from the control group (3, 3.13 %) (Table 3).

### The newborn conditions and follow-up

The average and median newborn birthweight were 3,429.02/3,435 g in the Omicron group, and 3,351.77/3,370 g in the control group, representing no significant difference (Table 1). Within three days of birth, 18 (39.13 %) newborns from the Omicron group were tested for SARS-CoV-2. Of these, 6 (33.33 %) tested positive, and 5 (83.33 %) were symptomatic. All symptomatic neonates developed a fever (5, 100 %), and one was diagnosed with pneumonia (20 %). The majority of newborns in the COVID-19 cohort achieved an Apgar score 9–10 (44, 95.65 %). Two years follow-up suggested that the Omicron-infected maternities and their newborns have no recent adverse outcomes.

## Discussion

In this retrospective analysis, we demonstrated that third-trimester Omicron infection induces a significant maternal systemic inflammatory response – characterized by fever and lymphopenia – and increased the risk of fetal distress. However, it did not result in severe specific placental pathology or adverse long-term neonatal outcomes. Consistent with reports on the general population, the majority of Omicron-infected pregnant women in our cohort experienced a mild clinical course, predominantly presenting with fever and lymphopenia. While lymphopenia is a known marker of viral infection, in the context of pregnancy, it underscores the distinct immune challenge imposed by the virus [13], [14], [15]. However, contrary to previous reports indicating that ancestral SARS-CoV-2 variants could lead to severe adverse pregnancy outcomes, our study did not observe major maternal complications or vertical transmission to the fetus [16].

A critical finding of our study was the significantly elevated incidence of fetal distress in the Omicron group compared to previously published data [17]. Despite this increased acute intrapartum risk, it did not lead to severe neonatal morbidity or mortality. This outcome highlights a critical clinical implication: the observed fetal distress likely reflects an acute physiological response to maternal systemic inflammation rather than irreversible fetal hypoxic injury. Timely obstetrical intervention effectively prevented the translation of intrauterine distress into severe neonatal outcomes. Additionally, while the gestational age at delivery was slightly lower in the Omicron group, the difference was not statistically significant, suggesting a trend toward earlier intervention rather than spontaneous preterm labor driven by infection [18].

The favorable clinical outcomes are biologically supported by our placental pathology findings. Existing literature suggests that acute inflammatory responses can impair placental vascular function, leading to adverse outcomes such as stillbirth or growth restriction [19]. However, our histological analysis revealed that features of maternal and fetal inflammatory responses, as well as MVM, occurred at statistically similar rates in both the Omicron and uninfected control groups. The absence of specific, severe placental destruction, coupled with normal fetoplacental weight ratios [15], 20], 21], suggests that the Omicron variant does not exhibit strong placental tropism or induce the extensive localized tissue damage seen with earlier variants.

Regarding neonatal transmission [22], [23], [24], while a small subset of tested newborns in our cohort was positive for SARS-CoV-2 and exhibited transient symptoms, all infants remained healthy at the two-year follow-up. This underscores that the infection did not result in long-term developmental or physical detriments. However, conclusively differentiating between true *in utero* vertical transmission and immediate postnatal environmental exposure remains challenging due to the high transmissibility of the Omicron variant.

A limitation of our study is the lack of detailed lineage sequencing prevents us from assessing the specific pathogenicity of distinct Omicron sub-variants (BA.5.2 vs. BF.7) during the study period. Additionally, the rapidly changing public health policy resulted in a non-universal screening strategy for newborns, which introduced a significant selection bias, hindering our ability to accurately assess the true incidence of vertical transmission. Furthermore, while our multivariable logistic regression identified Omicron infection as an independent risk factor for fetal distress, the resulting 95 % CI is wide due to the small sample size. This reflects potential model instability driven by low event rates, indicating that while the risk is significant, its precise magnitude remains uncertain. Finally, our entire study population consisted of women who had received three doses of a COVID-19 vaccine. The mild maternal symptoms, lack of severe complications, and favorable placental and neonatal outcomes observed in our cohort are likely mediated by vaccine-induced immunity. Consequently, these outcomes cannot be directly extrapolated to unvaccinated pregnant populations, who may face a significantly higher risk of severe disease.

## Conclusions

Our investigation into the effects of the SARS-CoV-2 Omicron variants on pregnancy outcomes involved a comprehensive two-year follow-up of 46 infected and 96 uninfected mothers. Our key findings highlighted an increased incidence of fetal distress among Omicron-infected mothers. The omicron group has a lower lymphocyte range and a higher CRP concentration than the control group. Effect of Omicron on placental pathology is not specific. Our two-year follow-up data revealed no long-term adverse outcomes in either the mothers or their newborns. Future research incorporating expanded clinical cohorts and prolonged observation periods is necessary to validate these findings and yield more conclusive insights.
